# Sleep habits and pattern in 1-14 years old children and relationship with video devices use and evening and night child activities

**DOI:** 10.1186/s13052-016-0324-x

**Published:** 2017-01-13

**Authors:** Paolo Brambilla, Marco Giussani, Angela Pasinato, Leonello Venturelli, Francesco Privitera, Emanuele Miraglia del Giudice, Sara Sollai, Marina Picca, Giuseppe Di Mauro, Oliviero Bruni, Elena Chiappini, on behalf of the “Ci piace sognare” Study Group, Salvatore Barberi, Sergio Bernasconi, Gianni Bona, Guido Brusoni, Carmen Buongiovanni, Marco Carotenuto, Mattia Doria, Daniele Ghiglioni, Manuel Gnecchi, Lorenzo Iughetti, Claudio Maffeis, Paola Manzoni, Maura Sticco, Gianni Tamassia, Elvira Verduci

**Affiliations:** 1 Family Pediatrician, Azienda Tutela della Salute (ATS) Città Metropolitana di Milano, Via Parada 32, 20854 Vedano al Lambro, MB Italy; 2 Family Pediatrician, ULSS 6 Vicenza, Vicenza, Italy; 3 Family Pediatrician, Azienda Tutela della Salute Bergamo, Bergamo, Italy; 4 Family Pediatrician, Azienda Sanitaria Provinciale Catania, Catania, Italy; 5 Dipartimento della Donna e del Bambino, Seconda Università di Napoli, Napoli, Italy; 6 AOU Meyer, Department of Health Science, Università di Firenze, Florence, Italy; 7 Family Pediatrician, Azienda Sanitaria Locale Caserta, Caserta, Italy; 8 Neuropsichiatria Infantile, Università della Sapienza, Rome, Italy

**Keywords:** Sleep duration, Sleep recommendations, Sleep continuity, Video devices, Bottle use, Bedroom TV, Body Mass Index

## Abstract

**Background:**

Sleep in childhood and adolescence is crucial for mental and physical health; however several researches reported an increasing trend towards a sleep deprivation in this age. Due to the lack of recent epidemiological studies in Italy, the aim of our study was to depict sleep habits and patterns in Italian children aged 1–14 years and to evaluate their relationships with video devices use (TV, tablet, smartphone, PC) and evening/night child activities.

**Methods:**

A structured interview was conducted during 2015 by 72 Family Pediatricians in 2030 healthy children aged 1–14 years by a cross-sectional survey named “Ci piace sognare”. Total sleep duration was calculated, 2015 National Sleep Foundation Recommendations were used as reference. Optimal sleepers were defined children sleeping in own bed all night without awakenings. Multivariable median regression was performed to identify predictors of sleep duration and multivariable logistic regression for predictors of optimal sleep.

**Results:**

Total sleep duration and numbers of awakenings decreased with age. Only 66.9% of children had sleep duration in agreement with Recommendations (50% in 10–14 years group). Before sleeping 63.5% of children used video devices (39.6% at 1–3 years), 39.1% read, 27.5% drank and 19.5% ate. Bottle users at bedtime were 30.8% at 1–3 years, 16.6% at 3–5 years and 4.9% at 5–7 years. Overall, 23.4% of children changed sleeping place during the night, 22.4% referred sleeping problems in the first year of life.

Video devices use was negative predictor of sleep duration (-0.25 h [95%CI:-0.35,-0.14], *p* < 0.001). Optimal sleep was inversely related with bedroom TV (OR 0.63 [0.50,0.79], *p* < 0.001), with sleeping disorders in the first year (OR 0.62 [0.48,0.80], *p* < 0.001)), with bottle use (OR 0.64 [0.44,0.94], *p* < 0.05) and posivively related with high mother’s education level (OR 1.44 [1.11,1.88], *p* < 0.01).

**Conclusions:**

About one third of 1 to 14 year Italian children sleep less than recommended, one half in teenage. Modifiable risk factors for sleep abnormalities such as video devices use, bedroom TV and bottle use should be target of preventive strategies for a correct sleep. Pediatricians should give priority to the identification of sleep disorders early in life.

**Electronic supplementary material:**

The online version of this article (doi:10.1186/s13052-016-0324-x) contains supplementary material, which is available to authorized users.

## Background

Sleep in childhood and adolescence is important for mental and physical health, as assessed by various papers in the last decade. Researches have shown that insufficient sleep is associated with obesity, metabolic risk, lower academic performance and emotional/behavior problems [1–4]. At the same time some studies have reported a reduction of sleep duration in pediatric ages [5, 6], thus suggesting to pediatricians the need for increasing their attention on this topic.

Empirical data demonstrated that several dimensions of sleep are related to health outcomes, and can be measured with self-report and objective methods, i.e., sleep duration, continuity and architecture [7]. Sleep duration and continuity (i.e., night awakenings) were the parameters more frequently studied in pediatric age.

Variables associated with short sleep duration in childhood have been proposed (latitude, cultural factors, late bedtime, etc.). In particular, a relationship between inadequate sleep and TV viewing and/or TV in the bedroom has been found by cross-sectional studies [8, 9]. Also more recent longitudinal studies showed a negative impact of daily TV viewing and use of other video devices (tablet, smartphone, PC) on sleep duration [10, 11]. TV viewing may directly displace bedtime or increase child emotional arousal and light exposure, all these mechanisms affecting sleep onset and duration [12]. However, few studies have investigated the impact of new devices (PC; tablet, smartphone, social network) on sleep quality.

Due to the lack of studies evaluating specifically all the evening activities at bedtime, aim of our study was to depict the sleep habits and the sleep patterns in a large national population of children aged 1–14 years and to evaluate their relationship with evening/night child activities.

## Methods

### Study design

Between April 2015 and November 2015 a cross sectional survey “Ci Piace Sognare” (CPS; literally: “We like dreaming”) was conducted among parents/caregivers of children aged 1 to 14 years referring to a group of Italian Family Paediatricians (FP) members of two Italian Pediatric Societies (Società Italiana di Pediatra Preventiva e Sociale and Società Italiana delle Cure Primarie Pediatriche).

The study was proposed in 2013 by the principal investigator (PB) to Scientific Board of the two Italian Pediatric Societies. A specific website was prepared for the puropse of the study.

### Family pediatricians

The study was announced during the Annual Meetings of both Societies yield in 2014. The participation of FP to the study was voluntary. Interested FPs were asked to register on the study website at beginning of 2015.

### Subjects

FPs were asked to enroll a maximum number of 2 children per day presenting in their office for a routine health visit and with the following characteristics: 1) age >1.0 and <14.0 years, 2) absence of any acute illness able to interfere with sleep. Children having parents with a poor command of the Italian language were excluded as well as children having any chronic disease able to interfere with sleep: celiac disease, diabetes, mucoviscidosis, cancer, chronic nephropathy, cardiopathy with hemodynamic impairment, syndrome with malformation, uncontrolled asthma, obstructive sleep apnea syndrome, neurological and neuropsychiatric disease (including autism and mental disability).

### Study design

A written informed Consensus was achieved by FPs from at least one of parents of each participant. The study was approved by Ethical Committee of Azienda Ospedaliero Universitaria “Maggiore della Carita” of Novara on 2^nd^ March 2015.

### Structured interview

The structured interview was elaborated by the Working Group (by adapting other existing and validated questionnaires) [13, 14] and contains questions on:

1. *child data* (birth date, gender, actual weight and height)
2. *family data* (age, job and education level of both parents, number of family members living with the child, number of brothers/sisters).
3. *sleep habits, pattern and bedtime/night environment during the last night* (time of falling asleep and of waking up, night sleep duration, naps and duration of daytime sleep, mean number of awakenings per night, place of falling asleep and of sleeping for the most part of the night, dinner time, foods and/or drinks before sleeping and during the night, bottle use, use of video devices (TV; PC; tablet, smartphone, etc.) just before sleeping), child use of active or passive reading before sleeping, presence of TV or other screen in child’s bedroom, presence of sleep problems during the first year of life, use of product for sleeping in the past or at study time.

The structured interview was prepared as a specific form to be fulfilled online anonymously (closed format questions) on the website by previously registered and trained FPs in the presence of at least one parent, in a weekday (Tuesday to Friday) during the period from 1^st^ April to 30 November 2015 (excluding school holidays and summertime).

A copy of the structured interview can be requested by mail to the corresponding author.

### Sleep items during the last night

Time of falling asleep and of waking up were approximated to 15 min (i.e., 21.15; 21.30; 21.45; 22.00, etc.), as well as dining time. Nocturnal sleep and daytime sleep duration were registered. Total sleep duration was calculated as the sum of nocturnal plus daytime sleep. Adequacy of total sleep duration was assessed by comparison with age-specific recommendations [15].

The place where the child fall asleep as well as where he/she slept for the most part of the night was registered among these options: own bed, parental bed, other room, outside the house. The presence of an own room, eventually shared with brothers/sisters, was investigated.

Drinks or foods consumed in 30-min interval before falling asleep or during the night was considered. The use of a device before sleeping were considered when it happened in the 30-min period before falling asleep.

The presence of TV in the room where the child usually sleeps, reading (active or passive) before sleeping and the history of sleep problems during the first year of life were investigated by means of closed answers. The use of products for sleeping in the past or at study time was investigated by means of multiple choice answers.

### Optimal sleepers

Children were classified as “optimal sleepers” if all the following conditions were present: 1) place of falling asleep: own bed; 2) place of sleeping: own bed, 3) no use of product for sleeping at study time; and 4) number of awakening equal to 0 (≤1 for children under 3 years of age). All other children were classified as “not optimal sleepers”.

### Child data

Pediatricians measured child weight and height (length up to 2 years of age) in the same day in which the interview was administered, using standard anthropometric procedures [16].

Body Mass Index (BMI) was calculated as weight (kg)/height (m^2^). BMI-Standard Deviation Score (SDS) as well as birth weight-SDS were calculated according with World Health Organization (WHO) Reference Tables, overweight and obese children were defined according with WHO BMI percentiles [17].

### Family data

Age, job and education level of both parents, number of family members living with the child, and number of brothers/sisters were investigated by means of closed format answers.

## Statistical analysis

Most continuous variables were not Gaussian-distributed and all are reported as 50^th^ percentile (median) and interquartile range (IQR) (25^th^ and 75^th^ percentiles). Discrete variables are reported as the number and percentage of subjects with the characteristic of interest. Descriptive data were reported by 5 age groups: 1 to <3, 3 to <5, 5 to <7, 7 to <10 and 10 to <14 years. Univariable median regression and univariable logostic regression were used to quantify the association of continuous and binary outcomes with the predictors of interest [18, 19]. The response variable of the median regression models was *total sleep* (hours) and that of the logistic regression models was *optimal sleeper* (0 = no, 1 = yes). Multivariable median regression was performed with the following prespecified predictors: 1) age (years), 2) BMI (SDS), 3) presence of TV in the bedroom, 4) use of display devices before sleeping, 5) drinking before sleeping. The multivariable logistic regression model had the following additional predictors: 6) high school or university degree of the mother, 7) personal room, 8) reading before sleep, 9) being only child, 10) mother working at home, 11) bottle use, and 12) sleep problems during the first year of life. Univariable and multivariable fractional polynomials were used to test whether the relationship between the response variable and the continuous predictors was linear [20]. All relationships were to be linear and were modelled as such. Multivariable quantile regression was used to estimate the 5^th^, 25^th^, 50^th^, 75^th^ and 95^th^ percentiles of total sleep by age and sex. Total sleep (hours) was used as the response variable and age (continuous, years) and sex (discrete: 0 = female; 1 = male) as predictors. Multivariable fractional polynomials of degree 2 were used to select transformations linearizing the sleep-age relationship [20]. Such transformations were age^-1^ for the 5^th^ percentile, age^0.5 for the 25^th^, 50^th^ and 95^th^ percentiles, and loge(age) for the 75^th^ percentile.

Statistical analysis was performed using Stata 14.1 (Stata Corporation, College Station, TX, USA).

## Results

The Working Group verified at study start that the 3 Italian macro-regions (North, Centre, South) were represented according with the known distribution of children under 14 years living in the country [21]. A hundred and one FPs expressed their interest to participate to CPS Study and 72 of them (71%) collected data, for a total of 2030 children. The median [IQR] number of children enrolled by each FP was 32 (28, 50).

Children characteristics are summarized in Table 1, stratified in the 5 age groups. Only in 2 cases parents refused to participate.

**Table 1:** Clinical characteristics of 2030 studied children according with age groups

	1 to 3 years			3 to 5 years			5 to 7 years			7 to 10 year			10 to 14 years			All subjects		
*n*	523			429			425			355			298			2030		
Males (%)	50.9			49.7			49.4			51.8			51.7			50.6		
N/C/S	252/92/179			182/80/167			189/63/173			164/57/134			173/49/76			960/341/729		
	*P50*	*P25*	*P75*	*P50*	*P25*	*P75*	*P50*	*P25*	*P75*	*P50*	*P25*	*P75*	*P50*	*P25*	*P75*	*P50*	*P25*	*P75*
Weight (kg)	11.8	10.5	13.0	16.0	14.7	17.9	21.0	19.0	24.0	28.9	25.0	33.6	43.0	35.5	52.0	19.0	14.0	28.5
BMI (kg/m)	16.2	15.4	17.2	15.7	14.8	16.7	15.7	14.7	17.1	16.7	15.3	18.9	18.6	17.0	21.5	16.3	15.2	17.9
BMI (SDS)	0.27	-0.43	0.98	0.28	-0.39	0.98	0.29	-0.40	1.11	0.44	-0.34	1.42	0.53	-0.34	1.36	0.32	-0.39	1.12

1 to 3 years: from 1.0 to 2.99 years; 3 to 5 years: from 3.0 to 4.99 years (similarly for other age groups)

*n* number of children

*N* North, *C* Centre, *S* South

*BMI* Body Mass Index

*SDS* Standard Deviation Score

*P50, P25* and *P75* represent median, 25^th^ and 75^th^ percentile

Median age was 5.25 years (IQR 5.12); 1027 of children (50.6%) were males, 960 (47%) lived in North Italy, 341 (17%) in Centre Italy and 729 (36%) in South Italy. The median (95%CI) BMI-SDS of children was 0.22 (0.13 to 0.32) in North Italy, 0.40 (0.25 to 0.56) in Centre Italy and 0.51 (95%CI 0.40 to 0.62) in South Italy. Overweight plus obese children were 27.3% in the overall population (21.8% at North, 29.1% at Centre, and 33.3% at South).

Seventy-seven percent of parents had high school or university education level with regional differences (83% North, 85% Centre and 66% South). Regional differences were observed also for mother’s job: employed mothers were 75% at North, 72% at Centre and 42% at South.

Drinks or foods consumed before sleeping or at night time were reported in Table 2. The most frequent dining time was 7.30 P.M. at North and 8.00 P.M. at Centre and South. Overall, 28.2% of children drank before sleeping, especially at younger ages, mostly milk. Twenty percent of children ate before sleeping regardless to age, mostly sweets. During the night drinking or eating was markedly less frequent: 4.7% and 1.1%, respectively. The bottle use at bedtime was 30.8% at 1 to 3 years, 16.6% at 3 to 5 years and 4.9% at 5 to 7 years.

**Table 2:** Drinks and foods consumed before sleeping or during the night in the study population

	Age groups (yrs)										All subjects	
	1 to 3		3 to 5		5 to 7		7 to 10		10 to 14			
	*N*	*%*	*N*	*%*	*N*	*%*	*N*	*%*	*N*	*%*	*N*	*%*
Subjects	523	100.0	429	100.0	425	100.0	355	100.0	298	100.0	2030	100.0
Drinks before sleep												
Nothing	270	51.6	283	66.0	334	78.6	302	85.1	268	89.9	1457	71.8
Milk	214	40.9	94	21.9	51	12.0	23	6.5	14	4.7	396	19.5
Juice	27	5.2	38	8.9	25	5.9	19	5.4	9	3.0	118	5.8
Milk & juice	8	1.5	5	1.2	4	0.9	3	0.8	2	0.7	22	1.1
Other	2	0.4	3	0.7	7	1.6	6	1.7	5	1.7	23	1.1
Unknown	2	0.4	6	1.4	4	0.9	2	0.6	0	0.0	14	0.7
Eats before sleep												
Nothing	418	79.9	342	79.7	346	81.4	280	78.9	236	79.2	1622	79.9
Fruit	25	4.8	15	3.5	12	2.8	12	3.4	10	3.4	74	3.6
Sweet	41	7.8	47	11.0	38	8.9	47	13.2	29	9.7	202	10.0
Salted	13	2.5	15	3.5	11	2.6	7	2.0	11	3.7	57	2.8
Fruit & sweet	2	0.4	2	0.5	4	0.9	0	0.0	1	0.3	9	0.4
Salted & sweet	4	0.8	2	0.5	1	0.2	1	0.3	1	0.3	9	0.4
Fruit & salted	7	1.3	0	0.0	2	0.5	3	0.8	4	1.3	16	0.8
Other	10	1.9	5	1.2	6	1.4	4	1.1	4	1.3	29	1.4
Unknown	3	0.6	1	0.2	5	1.2	1	0.3	2	0.7	12	0.6
Drinks during night												
Nothing	454	86.8	403	93.9	418	98.4	350	98.6	296	99.3	1921	94.6
Milk	55	10.5	13	3.0	4	0.9	1	0.3	0	0.0	73	3.6
Juice	8	1.5	8	1.9	1	0.2	2	0.6	0	0.0	19	0.9
Milk & juice	1	0.2	2	0.5	0	0.0	0	0.0	0	0.0	3	0.1
Other	1	0.2	1	0.2	0	0.0	0	0.0	1	0.3	3	0.1
Unknown	4	0.8	2	0.5	2	0.5	2	0.6	1	0.3	11	0.5
Eats during night												
Nothing	509	97.3	426	99.3	419	98.6	346	97.5	290	97.3	1990	98.0
Fruit	4	0.8	0	0.0	0	0.0	2	0.6	4	1.3	10	0.5
Sweet	2	0.4	1	0.2	0	0.0	1	0.3	0	0.0	4	0.2
Salted	0	0.0	0	0.0	0	0.0	1	0.3	0	0.0	1	0.1
Fruit & sweet	1	0.2	0	0.0	0	0.0	0	0.0	1	0.3	2	0.1
Other	3	0.6	1	0.2	0	0.0	0	0.0	0	0.0	4	0.2
Unknown	4	0.8	1	0.2	6	1.4	5	1.4	3	1.0	19	0.9
Bottle use before sleep	161	30.8	71	16.6	21	4.9	2	0.6	4	1.3	259	12.8

Sleep variables were reported in Table 3. Total sleep decreased with age from 11.5 h (1.5) (median (IQR)) in 1 to 3 years old children to 9.0 h (1.25) in 10 to 14 years old. Daytime sleep was negligible after 5 years of age. Overall, 1358 children (66.9%) had a total sleep duration in agreement with the NSF 2015 recommendations. Such proportion varied between 64 and 77% up to 10 years of age and dropped to 50% thereafter. Sleep duration was shorter than recommended in 642 children (31.6%) and longer than recommended in 30 children (1.5%). Children living at South (62.4%) and at Centre (66.9%) followed recommendations in lower percentages than those living at North (72.6%).

**Table 3:** Sleep duration and sleep related characteristics in the study population

	Age groups (yrs)										All subjects	
	1 to 3		3 to 5		5 to 7		7 to 10		10 to 14			
Subjects	523	100.0	429	100.0	425	100.0	355	100.0	298	100.0	2030	100.0
	*Med*	*IQR*	*Med*	*IQR*	*Med*	*IQR*	*Med*	*IQR*	*Med*	*IQR*	*Med*	*IQR*
Nocturnal sleep (hrs)	*9.50*	*1.25*	*9.75*	*1.25*	*9.75*	*1.00*	*9.50*	*0.75*	*8.88*	*1.25*	*9.50*	*1.00*
Daytime sleep (hrs)	2.00	1.00	0.75	1.50	0.00	0.00	0.00	0.00	0.00	0.00	0.00	1.50
Total sleep (hrs)	11.50	1.50	10.50	1.00	9.75	1.00	9.50	1.00	9.00	1.25	10.00	1.75
	*N*	*%*	*N*	*%*	*N*	*%*	*N*	*%*	*N*	*%*	*N*	*%*
Sleep duration according with Recommendations^a^												
Under	171	32,7	104	24.2	141	33.2	82	23.1	144	48.3	642	31.6
Recommended	346	66.2	319	74.4	272	64.0	272	76.6	149	50.0	1358	66.9
Over	6	1.1	6	1.4	12	2.8	1	0.3	5	1.7	30	1.5
Awakenings												
0	170	32.5	204	47.6	294	69.2	272	76.6	223	74.8	1163	57.3
1	156	29.8	144	33.6	96	22.6	60	16.9	52	17.4	508	25.0
2	120	22.9	46	10.7	31	7.3	14	3.9	18	6.0	229	11.3
3	39	7.5	27	6.3	3	0.7	6	1.7	4	1.3	79	3.9
≥4	38	7.3	8	1.8	1	0.2	2	0.6	1	0.3	50	2.4
Unknown	0	0.0	0	0.0	0	0.0	1	0.3	0	0.0	1	0.0
Owns a room												
Yes	212	40.5	234	54.5	211	49.6	181	51.0	164	55.0	1002	49.4
No	209	40.0	90	21.0	48	11.3	27	7.6	7	2.3	381	18.8
Shared	97	18.5	100	23.3	166	39.1	146	41.1	127	42.6	636	31.3
Unknown	5	1.0	5	1.2	0	0.0	1	0.3	0	0.0	11	0.5
Has TV in bedroom	131	25.0	155	36.1	182	42.8	184	51.8	158	53.0	810	39.9
Display devices use	207	39.6	281	65.5	295	69.4	269	75.8	237	79.5	1289	63.5
Reads before sleep	189	36.1	208	48.5	183	43.1	120	33.8	93	31.2	793	39.1
Where falls asleep												
Own bed	245	46.8	218	50.8	264	62.1	255	71.8	260	87.2	1242	61.2
Parents’ bed	203	38.8	157	36.6	109	25.6	70	19.7	23	7.7	562	27.7
Other room	67	12.8	48	11.2	49	11.5	28	7.9	15	5.0	207	10.2
Outside home	5	1.0	5	1.2	2	0.5	2	0.6	0	0.0	14	0.7
Unknown	3	0.6	1	0.2	1	0.2	0	0.0	0	0.0	5	0.2
Where sleeps												
Own bed	363	69.4	284	66.2	333	78.4	289	81.4	278	93.3	1547	76.2
Parents’ bed	157	30.0	137	31.9	84	19.8	61	17.2	17	5.7	456	22.5
Other room	2	0.4	5	1.2	6	1.4	3	0.8	2	0.7	18	0.9
Outside home	0	0.0	2	0.5	1	0.2	2	0.6	0	0.0	5	0.2
Unknown	1	0.2	1	0.2	1	0.2	0	0.0	1	0.3	4	0.2

^a^NSF recommendations (ref. [15])

**Fig. 1:**
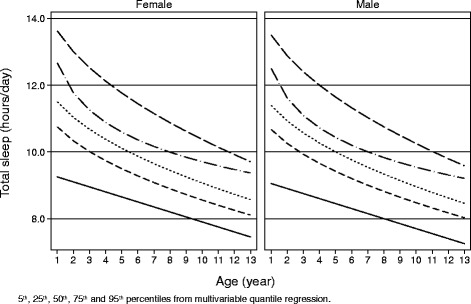
Percentiles of total sleep duration as function of age and gender in the study population

Figure 1 plots the percentiles of sleep duration as function of age in the whole sample (*n* = 2030). Such percentiles were estimated from quantile regression (see Statistical analysis for details).

Table 3 reported the number of night awakenings divided by age: specifically no awakenings were reported in 32.5% in 1–3 years group and increased to 74.8% in 10–14 years group; more than 2 awakenings were reported 14.8% in the younger group and decreased to 1.6% in the older group.

Overall, 63.5% of children used video devices (39.6% at age 1–3, increasing thereafter till 79.5% at age 10–13) and 39.1% read before sleeping (with a maximum of 48.5% at age 3–5 and then a progressive decrease). Overall, 61.2% of children fell asleep in their own bed, 27.7% in parents’ bed and 10.2% in other room. Children falling asleep in their bed increased with age and those falling asleep in parents’ bed decreased. Children sleeping in their own bed for the most part of the night increased with age from 69,4% at 1 to 3 years to 93.3% at 10 to 14 years. Overall, 23.4% of children changed place of sleeping during the night, mostly from parents’ to own bed (10.1%), from other room to own bed (7.6%) or from own to parents’ bed (3.0%).

Use of products for sleep were reported in Table 4. At study time 2.1% of parents reported use of products for sleeping, while 10.0% reported its use in the past. In 22.4% of children an history of sleeping problems in the first year of life was found.

**Table 4:** Use of products for sleeping in the study population

	Age groups (yrs)										All subjects	
	1 to 3		3 to 5		5 to 7		7 to 10		10 to 14			
	*N*	*%*	*N*	*%*	*N*	*%*	*N*	*%*	*N*	*%*	*N*	*%*
Subjects	523	100.0	429	100.0	425	100.0	355	100.0	298	100.0	2030	100.0
Sleep disorder in 1st year												
No	342	65.4	302	70.4	317	74.6	267	75.2	209	70.1	1437	70.8
Yes	136	26.0	98	22.8	88	20.7	67	18.9	65	21.8	454	22.4
Sometimes	44	8.4	29	6.8	20	4.7	21	5.9	21	7.0	135	6.7
Unknown	1	0.2	0	0.0	0	0.0	0	0.0	3	1.0	4	0.2
Has used products to sleep												
No	462	88.3	391	91.1	390	91.8	316	89.0	266	89.3	1825	89.9
Yes	59	11.3	38	8.9	35	8.2	39	10.7	32	10.7	202	10.0
Unknown	2	0.4	0	0.0	0	0.0	0	0.3	0	0.0	3	0.1
Has used:												
Melatonin	35	6.7	23	5.4	17	4.0	14	3.9	8	2.7	97	4.8
Phytotherapy	19	3.6	14	3.3	13	3.1	15	4.2	9	3.0	70	3.4
Tryptophan	2	0.4	1	0.2	0	0.0	1	0.3	2	0.7	6	0.3
Vitamin B6	3	0.6	2	0.5	0	0.0	1	0.3	0	0.0	6	0.3
Homeopathy	12	2.3	6	1.4	9	2.1	10	2.8	7	2.3	44	2.2
Other products	3	0.6	3	0.7	5	1.2	7	2.0	9	3.0	27	1.3
Suggested by:												
Pediatrician	47	9.0	32	7.5	27	6.4	32	9.0	24	8.1	162	8.0
Other parents	2	0.4	1	0.2	0	0.0	1	0.3	0	0.0	4	0.2
Pharmacist	9	1.7	5	1.2	4	0.9	6	1.7	3	1.0	27	1.3
Auto-prescription	2	0.4	1	0.2	5	1.2	2	0.6	6	2.0	16	0.8
Are products to sleep effective												
No	20	3.8	11	2.6	14	3.3	10	2.8	11	3.7	66	3.3
Sometimes	26	4.8	20	4.7	14	3.3	17	4.8	8	2.7	84	4.1
Yes	13	2.5	7	1.6	7	1.6	12	3.4	13	4.4	52	2.6
Uses products to sleep now												
No	502	96.0	420	97.9	421	99.1	352	99.2	291	97.7	1986	97.8
Yes	21	4.0	9	2.1	3	0.7	3	0.8	7	2.3	43	2.1
Unknown	0	0.0	0	0.0	1	0.2	0	0.0	0	0.0	1	0.0

Table 5 reports sleep related variables observed in optimal sleepers (752 children, 37.0%) and not optimal sleepers (1278 children, 63.0%), as defined in Methods section. Mean age (7.1 year [5.0, 10.0] (median [IQR]) was higher in optimal sleepers than in not optimal sleepers (4.0 year [2.3, 6.3]), while total sleep duration was lower (9.5 h [9.0, 10.2] vs 10.2 h [9.5, 11.2]). BMI SDS was similar in both groups (optimal sleepers 0.34 [-0.40, 1.19], not optimal sleepers 0.31 [-0.38, 1.08]).

**Table 5:** Sleep related variables observed in optimal sleepers and not optimal sleepers

	Not optimal sleepers (*n* = 1278)		Optimal sleepers (*n* = 752)	
	*N*	%	*N*	%
Child				
Male	627	49.1	400	53.2*
Normal weight	704	67.8	490	68.8
Firstborn	754	59.0	450	59.8
Only child	490	38.3	158	21.0*
Family				
Mother’edu: high school or univ	964	75.4	606	80.6*
Mother employed	780	61.0	487	64.8*
Father’s edu: high school or univ	893	69.9	522	69.4
Father employed	1164	92.8	684	92.1
Sleep items				
Follows NSF recommendations	872	68.2	508	67.6
Has TV in bedroom	523	41.1	287	38.3*
Display devices use before sleeping	783	61.5	506	67.3*
Reads before sleep	486	38.2	307	40.9*
Sleep problems during the 1^st^ year	325	25.5	129	17.2*
Has used products to sleep in the past	151	11.8	51	6.8*
Drink & food items				
-Drinks before sleep:				
Nothing	848	66.8	609	81.5*
Milk	311	24.5	85	11.4*
Juice	84	6.6	34	4.6*
-Bottle use	218	17.2	41	5.5*
-Eats before sleep:				
Nothing	1012	79.7	610	81.4
Fruit	53	4.2	21	2.8
Sweet	119	9.4	83	11.1
Salted	42	3.3	15	2.0
-Eats during night:				
Nothing	1250	98.7	740	99.3
-Drinks during night:				
Nothing	1178	92.7	743	99.3*
Milk	70	5.5	3	0.4*
Juice	17	1.3	2	0.3*

*p value < 0.05 respect to not optimal sleepers

Optimal sleepers showed a higher proportion of males, their mothers had higher education level and were more frequently employed. Optimal sleepers were less frequently only child, used bottle, had TV in the bedroom, drank before sleeping, presented sleep disorders during the first year of life and used product for sleeping, while they read before sleeping more frequently. Percentages of children in agreement with 2015 NSF recommendations for sleep duration were similar in both groups.

### Predictors of total sleep duration

At multivariable median regression, (Table 6) an increase of 1 year of age was associated with a decrease of 0.24 h [95%CI -0.25 to -0.22, *p* < 0.001] of total sleep, the use of display devices before sleeping was associated with a decrease of 0.25 h [95%CI -0.35 to -0.14, *p* < 0.001]. Other significant predictors found at univariable level (i.e., having TV in the bedroom, and drinking before sleeping.) were no more associated with total sleep duration when age and display devices use were taken into account at multivariable level. BMI-SDS was not associated with total sleep duration (-0.02 h [-0.06, 0.02]), even in the obese group alone. Similarly no association was found with parental age, parental education or job, number of family members, birth weight, to be firstborn or only child, feel asleep in own bed, bottle use, reading or eating before sleeping, or the presence of sleep disorders in the first year of life.

**Table 6:** Multivariable median regression for total sleep duration

	Total sleep (hours)
Age (years)	-0.24*** [-0.25,-0.22]
BMI (SDS)	-0.02 [-0.06,0.02]
Has TV in bedroom	-0.08 [-0.18,0.03]
Use of display devices	-0.25*** [-0.35,-0.14]
Drinks before sleep	-0.06 [-0.13,0.00]
Constant	11.76*** [11.61,11.90]
Observations	2006

Multivariable median regression

Value are regression coefficients [95% CI]

***p < 0.001

**Table 7:** Multivariable logistic regression for optimal sleep condition

	Optimal sleep
Age (years)	1.28*** [1.23,1.32]
BMI (SDS)	1.00 [0.92,1.09]
Has TV in bedroom	0.63*** [0.50,0.79]
Use of display devices	0.91 [0.72,1.14]
Drinks before sleep	0.99 [0.85,1.14]
Mother has high school or university degree	1.44** [1.11,1.88]
Owns a room	1.06 [0.94,1.19]
Reads before sleep	1.17 [0.94,1.44]
Only child	0.60*** [0.47,0.78]
Mother works at home	0.95 [0.75,1.20]
Bottle use	0.64* [0.44,0.94]
Sleep disorder at < 1 year of age	0.62*** [0.48,0.80]
Observations	1977

Multivariable logistic regression. Values are odds ratios

Values are odds ratios [95% CI]

*p < 0.05, **p < 0.01, ***p < 0.001

### Predictors of optimal sleep

At multivariable logistic regression (Table 7), optimal sleeper condition was positively associated with age (OR 1.28 [1.23, 1.32], *p* < 0.001), and with mother’s high education level (OR 1.44 [1.11, 1.88], *p* < 0.01) and negatively associated with having TV in the bedroom (OR 0.63 [0.50, 0.79], *p* < 0.001), being only child (OR 0.60 [0.47, 0.78], *p* < 0.001), bottle use (OR 0.64 [0.44, 0.94], *p* < 0.05), and sleep disorders during the first year of life (OR 0.62 [0.48, 0.80], *p* < 0.001). Other significant predictors found at univariable level (i.e., use of display devices, drinking or before sleeping, own a room, or mother working at home) were no more associated with optimal sleep when the previous predictors were taken into account at multivariable level. BMI-SDS was not associated with good sleeping (OR 1.00 [0.92, 1.09]) as well as all other variables.

## Discussion

The main results of the present study was that 33.1% of 1 to 14 years old children did not follow sleep duration recommendations, and that the percentage dropped to 50% in teenage. Because the study used convenience sampling, its results should not be extrapolated to the general population. However, the large sample size and the regional distribution of studied children, very close to that known in Italy, let us suggest that these data could describe a real phenomenon, as nationalwide collected sleep data are still lacking in our country at present. Of the 33.1% of children not coping with recommendations, the great majority are referred to sleep less than the lower limit and only the 1.5% more than the upper limit of recommendations. Multivariable regression analysis indicated that the only independent factor associated with sleep duration was the use of a video device in the imminence of bedtime. A negative relationship between videotime and sleep has been already suggested by others studies [8–11, 22, 23], in adolescence but recently also in younger ages due to the widespread and earlier use of technology [24]. We found that the use of a video device close to bedtime in childhood was related to a short sleep independently of the presence of bedroom TV, and this fact might be explained by the increasing use of mobile devices [25]. Literature reports a relationship between light exposure from video devices at bedtime and melatonin suppression, suggesting a possible explaination for the linking between video use and sleep duration [12, 23, 24]. We acknowledge that it seems unfeasible to avoid any video dependence for children at present time, but the relationship between video use close to bedtime and short sleep should be stressed. Of note, we did not found any relationship between sleep duration and child BMI, in contrast with the prevalent literature on this topic [26, 27], but in accordance with others [28]. This discrepancy among different studies might be due to variables considered, as it is known that many factors (and video use above all) are related with both sleep and obesity status.

In our study we considered also sleep continuity, defining as optimal sleepers those children sleeping in their own bed without awakenings throughout the night. Number of awakenings was higher in younger age groups as well as number of children falling asleep or sleeping out of their own bed. We consider very impressive that about one fourth of children changed place of sleeping during the night. Children defined as optimal sleepers (globally the 37% of our population) were generally older but with a similar median BMI-SDS respect to not optimal ones, thus confirming the low impact of BMI status on sleep in our population. Multivariable regression analysis indicated that independent factors associated with optimal sleep condition were high mother’s educational level, being only child, an history of sleep problems during the first year of life, present bottle use and bedroom TV. Some of these findings deserve a specific discussion.

An early history of sleep problems affecting further sleep continuity suggests the importance of establishing a correct sleep pattern very soon after birth, taking into account that a relative stability of sleep characteristics has been described starting from 6 month of age [29]. Moreover, parents and pediatricians should give an extreme importance to prevent sleep problems from birth.

Pediatricians usually suggest bottle use weaning at or around 12 months of age, but this recommendation is greatly ignored [30]. In our population, 4.9% of 5 to 7 years children used bottle in the imminence of sleeping time, and the use decreased thereafter but was still detectable (0.6% at 7 to 10 years and 1.3% at 10 to 14 years). A prolunged bottle use seems to be related to an alteration of sleep pattern, at least for children under 3 years of age, as found by other studies [31]. The negative effect of bottle use on sleep continuity found in our analysis suggests that this relationship might be present also in older children and underlines the need for an identification and possibly correction of such neglected attitude in late bottle consumers.

Also bedroom TV was associated with not optimal sleep, and this confirmed previous finding of the negative impact of video devices on sleep [8, 10, 11].

No effect of bedtime reading, and drink or food consumption was found on sleep continuity or duration in our population, when previous reported variables were taken into account.

A strenght of the present study is the characterization of sleep duration percentiles for age and gender, specific for Italian population and useful in clinical practice, which are similar but not coincident with those already available from other Countries. For instances, sleep duration in Italian children seems to be shorter than that reported in English peers [32].

Among study limitations we should consider first of all the cross sectional design which does not allow to determine casuality between considered variables and sleep items. Moreover, the lack of sleep latency data among studied parameters limits the assessment of sleep quality in our population. Finally, we considered only video devices use in the imminence of sleeping and we did not collect information concerning daily video consume, thus making impossible any correction for that in the analysis.

A recent technical report of the American Academy of Pediatrics [33], analysing both benefits and risks of new media use on child health, stressed the negative impact of video use on sleep characteristics and suggested the adoption of an healthy Family Media Use Plan individualized for a specific child and family, in order to identify an appropriate balance between video time and other activities.

## Conclusion

In conclusion there is a consistent percentage of children and adolescent that do not sleep sufficiently and this sleep deprivation could lead to neurobehavioral dysfunction. Pediatricians and mainly family pediatricians should give relevance to the identification of sleep problems early in life and in particular acting on the modifiable risk factors identified in the present study like video use at bedtime, bedroom TV, bottle use before sleep. Furthermore the fact that an history of sleep problems during the first year is related to not optimal sleep later in the life highlights the importance of ensuring a good sleep since the first months of life adopting correct preventive strategies.

## Additional file

Additional file 1: Dataset. (XLS 4134 kb)

## Abbreviations

BMI: Body Mass Index
CI: Confidence interval
FP: Family Pediatricians
IQR: Inter quartile range
NSF: National Sleep Foundation
OR: Odds ratio
PC: Personal computer
SDS: Standard Deviation Score
TV: Television set
WHO: World Health Organization
